# Cyr61, a marker of progesterone activity in normal and hyperplasic endometrium of female dogs

**DOI:** 10.1186/1753-6561-7-S2-P22

**Published:** 2013-04-04

**Authors:** Fabiana A Voorwald, Caio F Tiosso, Carolina F João, Diogo J Cardilli, Renée L Amorim, Gilson H Toniollo

**Affiliations:** 1 Department of Clinical and Veterinary Surgery, FCAV/UNESP, Jaboticabal, SP, Brazil; 2 Federal University of Pará, UFPA, Castanhal, PA, Brazil; 3 Department of Preventive Veterinary Medicine and Animal Reproduction, FCAV/UNESP, Jaboticabal, SP, Brazil; 4 Department of Veterinary Sciences, FMVZ/UNESP, Botucatu, SP, Brazil

## Background

Cysteine-rich protein 61 (CYR61/CCN1) is a growth factor-inducible gene, involved in the regulation of multiple cellular processes, such as cell adhesion, migration, survival, growth factor-induced mitogenesis, endothelial tubule formation, apoptosis and angiogenesis. In normal women endometrium, CYR61 expression is elevated during the proliferative phase of the menstrual cycle, and reduced during the midsecretory phase. However, CYR61 expression is deregulated in endometrium with estrogen dependent diseases, such as endometriosis, polycystic ovarian syndrome, endometrial hyperplasia and adenocarcinoma.

## Materials and methods

CYR61 expression in endometrium of 20 prepubertal female dogs, 20 female dogs at each phase of the estrous cycle and 24 dogs affected by cystic endometrial hyperplasia associated with pyometra, was evaluated by immunohistochemistry, analyzed by Leica QWin Plus V.3.5.0 automated system and statistically analyzed and correlated with estrogen and progesterone plasmatic levels.

## Results

CYR61 protein was detected in glandular and luminal epithelial cells of endometrium. Prepubertal female dogs had significantly the lowest expression of Cyr61, followed by proestrus, estrus and anestrus phases. Patients affected by pyometra revealed significantly the highest staining area, followed by diestrus phase. Therefore, Cyr61 in normal endometrium was highest during the secretory phase and lowest during proliferative phase of the estrus cycle, resulting in significant positive linear correlation of cytoplasmatic staining area with progesterone levels. There was no significant correlation with estrogen levels.

## Conclusions

In female dogs, Cyr61 is overexpressed in hyperplasic endometrium affected by pyometra, a process mediated by progesterone and only aggravated by estrogens. Cyr61 low expression in prepubertal endometrium and proestrus phase, suggest that Cyr61 is not regulated by estrogen, but by progesterone responsiveness, which can be caused by specific characteristics of long luteal phase, characteristic of this species.

## Financial support

FAPESP.

